# Patient engagement in radiation oncology: a large retrospective study of survey response dynamics

**DOI:** 10.3389/fonc.2024.1434949

**Published:** 2025-01-17

**Authors:** Bailey A. Loving, Hong Ye, Elizabeth Rutka, John M. Robertson

**Affiliations:** Department of Radiation Oncology, Corewell Health William Beaumont University Hospital, Royal Oak, MI, United States

**Keywords:** patient engagement, satisfaction surveys, radiation oncology, socioeconomic status (SES), Area Deprivation Index (ADI), telemedicine, mental health disorders (MHD), patient-centered care

## Abstract

**Purpose:**

Patient satisfaction surveys are pivotal in evaluating healthcare quality and enhancing patient care. Understanding the factors influencing patient engagement with these surveys in radiation oncology can guide improvements in patient-centered care.

**Methods:**

This retrospective study analyzed data from radiation oncology patients at a large multi-site single-institution center from May 2021 to January 2024. We assessed the influence of demographic, clinical, and socioeconomic factors on the likelihood of survey participation using univariate (UVA) and multivariable (MVA) logistic regression analyses. Factors included age, gender, race, socioeconomic status (SES) via Area Deprivation Index (ADI), language, marital status, smoking, employment, insurance type, mental health disorders (MHD), comorbidity index (CCI), and cancer type.

**Results:**

In a comprehensive analysis of 11,859 patients, most were female (57.2%), over 65 years old (60.7%), and primarily insured by Medicare (45.9%). MVA showed that higher socioeconomic disadvantage significantly decreased survey participation (ADI third tertile vs. first tertile OR=0.708, p<0.001), with each unit increase in ADI reducing the odds of completion by 1% (p<0.001). Older adults, and patients with head and neck or genitourinary cancers were significantly more likely to participate, while those with higher comorbidities, MHD, or other minority status were less engaged (p<0.001). Telemedicine encounters also significantly increased participation compared to in-person visits (OR=1.149, p=0.006).

**Conclusions:**

Multiple factors including age, race, SES, insurance type, cancer type, health conditions, and modality of healthcare delivery influence patient engagement with satisfaction surveys in radiation oncology. Strategies to enhance patient engagement must consider these diverse influences to ensure comprehensive and inclusive feedback mechanisms in healthcare settings. Tailored interventions to mitigate barriers specific to underrepresented groups are crucial for capturing a broad spectrum of patient experiences and improving the overall quality of patient care.

## Introduction

### The pursuit of patient engagement in survey responses

Patient engagement is increasingly recognized as an integral part of healthcare, shaping the way services are designed, delivered, and managed. Bombard et al. (1) emphasize that involving patients in decision-making improves healthcare governance while enhancing health outcomes and patient care (1). Both Ramdurai (3) and Greene (2) highlight the practical benefits, linking deeper patient engagement to improved care and potential cost savings (2, 3). Greene et al. demonstrated that higher patient activation levels were associated with better health outcomes—such as improved clinical indicators and preventive care—and significantly lower healthcare costs over time (2). Patient activation refers to an individual’s health literacy, readiness to change, and confidence in managing their health, whereas patient engagement encompasses the broader process of involving patients in their care, fostering collaboration, and ensuring their preferences are reflected in healthcare decisions (4). Similarly, Ramdurai et al. highlighted that greater patient engagement can yield significant financial benefits, including reducing no-shows and canceled appointments, improving patient retention, and fostering revenue growth by maintaining the current patient base while attracting new patients (3). Integrating patients into care discussions fosters collaboration, leading to greater satisfaction as their needs and preferences are acknowledged (5, 6).

Patient satisfaction surveys directly impact outcomes like hospital profitability, lawsuit rates, compliance, safety culture, and readmission (7–13). These surveys highlight areas for improvement, fostering patient-centered care to enhance healthcare quality (14). At our institution, these surveys are reviewed quarterly by a multidisciplinary quality improvement committee, and action items are generated based on aggregate results to address areas needing improvement. Semiannual departmental meetings further ensure alignment of these initiatives with broader institutional goals.

The National Research Corporation (NRC), a leader in healthcare research, developed patient satisfaction surveys to evaluate and improve the quality of care by gathering actionable patient feedback. Our institution uses a 14-question NRC survey, adapted from the American Nurses Credentialing Center (ANCC) Magnet Recognition Program (15, 16). The ANCC, a globally recognized body for nursing excellence, emphasizes patient-centered practices that align with evidence-based care. These aspects were tailored to reflect the unique needs of radiation oncology patients and are distributed via email, Interactive Voice Response (IVR), or text message (SMS) to ensure broad reach (15, 17). The survey questions are included as **Supplementary Table 1**. Factors like socioeconomic status (SES), cancer type, insurance, race, gender, comorbidities, and mental health disorders (MHD) can affect survey participation, potentially skewing representation of certain groups. Our previous publication further examined these dynamics, offering detailed comparisons of survey responses by question with patient demographics, particularly in the context of socioeconomic disadvantage (18).

### Factors that impact patient engagement

Research on factors influencing patient engagement in satisfaction surveys remains limited, with only a few studies exploring psychometric elements affecting patient participation (19–22). Davis et al. (23) identified five categories potentially impacting patient involvement: patient demographics, illness severity, provider beliefs, care setting, and task complexity (23). However, these factors were not directly measured, being extrapolated from other treatment decision-making research and identified as common themes (23). Strickler et al. (24) found that disparities in response rates in emergency department patients persist across age, gender, and payor classification, when using electronic surveys, highlighting that electronic surveys alone may not effectively address these disparities (24). Gayet-Ageron et al. (25) linked non-participation to language barriers, substance use disorders, cognitive disorders as identified through positive minimal mental status or neuropsychological screenings during hospital stays, psychiatric diagnoses, and visual impairments (25).

Accurately representing the radiation oncology patient population is vital, as insights from patient satisfaction surveys can directly impact quality improvement initiatives. Despite the importance of this, no studies have examined differences between survey responders and non-responders in this field. Our study addresses this need through a large, retrospective analysis, exploring the distinctions between those who respond to satisfaction surveys and those who do not.

## Methods

### Study design and participants

This study was conducted at four outpatient academic radiation oncology centers within our institution, spanning from May 2021 to January 2024. NRC patient satisfaction surveys were administered prospectively to patients receiving care and were distributed via email, Interactive Voice Response (IVR), or text message (SMS). The surveys were available in multiple languages, including English, Spanish, Mandarin, Russian, Korean, Vietnamese, Hindi, and Arabic, among others. To avoid oversampling, patients who completed a survey during a consultation were not eligible to receive another survey for the next 120 days. The study received approval from the Institutional Review Board (IRB). Data from the Electronic Medical Records System (EMR) were extracted for every encounter involving patients who signed a general release form. Matching these data with the survey data allowed us to distinguish between patients who completed the surveys (responders) and those who did not (non-responders). Patients with incomplete demographic information or those under 18 or over 99 were excluded.

### Database query

We systematically collected a range of variables for both the responder and non-responder groups to ensure a comprehensive analysis. Patient characteristics included age, gender, race, preferred language, marriage status, employment status, smoking, insurance type, and Area Deprivation Index (ADI) to account for socioeconomic factors. Disease specifics included cancer type, history of MHD, Charlson Comorbidity Index (CCI), and mortality records. Detailed healthcare encounter information was also collected, such as encounter type (consult, follow-up, procedure, treatment), telemedicine use, and hospital site.

### Socioeconomic status measurement

To measure the SES of participants, we utilized the ADI, obtained from the Neighborhood Atlas (26, 27). The ADI correlates with the socioeconomic conditions of a geographic area by incorporating 17 U.S. Census variables across four primary domains: income, education, employment, and housing quality (26). Examples of these variables include the percentage of households below the poverty level, the percentage of adults without a high school diploma, the unemployment rate, and the percentage of overcrowded housing (26). Each patient’s 9-digit zip code was matched to the corresponding ADI, enabling a precise evaluation of their SES based on the specific characteristics of their residential location. The ADI provides both national percentiles and state deciles, categorizing patients by different levels of socioeconomic disadvantage. Higher scores denote greater disadvantage, allowing for a nuanced analysis of SES’s impact on patient engagement. For simplicity of the analysis and to ensure generalizability, only the national ADI was used.

### Charlson comorbidity index

The CCI assigns weights to comorbid conditions, predicting the ten-year mortality rate (28). Each condition is scored based on its associated mortality risk, with the scores summed to predict overall mortality. CCI has been validated for adjusting disease burden and mortality prediction (29, 30). In our study, relevant ICD-10 codes were collected from the EMR to calculate CCI scores, which were then integrated into the broader analysis framework.

### Statistical analysis

For our statistical analysis, we initially used Pearson’s chi-square test for categorical variables and the non-parametric Mann-Whitney U test for continuous variables to identify preliminary differences between survey responders and non-responders. A univariate logistic regression analysis (UVA) was then conducted to identify key predictors of survey response. Variables that demonstrated a near-significant trend on UVA (p < 0.05) were included in the initial multivariable logistic regression model (MVA).

To construct the MVA model, we employed a backward stepwise logistic regression model with a p-value threshold of 0.10 for variable removal. This approach allowed for the systematic exclusion of the least significant variables while maintaining the overall integrity and explanatory power of the model. Variance inflation factors (VIF) were calculated for all variables prior to their inclusion in the MVA to assess and address multicollinearity.

In a *post-hoc* analysis, Pearson’s chi-square tests were used for categorical data and Kruskal-Wallis tests for continuous data to confirm statistical differences across variables. Significance was defined at α = 0.01, with a near-significant trend noted at α = 0.05. All statistical analyses were conducted using Python (version 3.12.3) with the Pandas, NumPy, and SciPy libraries.

## Results

We identified a total of 12,058 patients from our multi-site institution who visited our department from May 2021 to January 2024. After excluding those with incomplete demographic data or outside the age range of 18 to 99 years, 11,859 patients remained eligible for analysis.

As outlined in **Table 1**, the cohort largely comprised females (57.2%), individuals over the age of 65 (60.7%), and patients with breast cancer (28.5%). Most patients identified as White or Caucasian (80.9%), with Medicare providing coverage for the largest group (45.9%). Most patients were retired (49.1%), never smokers (43.4%), primarily English-speaking (80.8%), and married (58.1%). Follow-up visits comprised 75.0% of all encounters, and 21.1% of all visits, including consults and follow-ups, were conducted via telemedicine. The majority had a CCI score below 4 (77.7%) and no history of an MHD diagnosis (84.5%). Additionally, 52.9% lived in areas with a national ADI ranking below the mean.

**Table 1:** Patient characteristics.

Characteristic	All Patients	Respondents	Non-Respondents	p value
Age >65				
No	4660 (39.3%)	1591 (31.2%)	3069 (45.5%)	<0.001
Yes	7187 (60.7%)	3514 (68.8%)	3673 (54.5%)	
Age (Continuous)	68.00 (60.00-75.00)	70.00 (62.00-76.00)	66.00 (58.00-74.00)	<0.001
Gender				
Female	6781 (57.2%)	2921 (57.2%)	3860 (57.3%)	0.970
Male	5066 (42.8%)	2184 (42.8%)	2882 (42.7%)	
Marital Status				
Divorced	1451 (12.2%)	593 (11.6%)	858 (12.7%)	<0.001
Married	6879 (58.1%)	3094 (60.6%)	3785 (56.1%)	
Single	1990 (16.8%)	744 (14.6%)	1246 (18.5%)	
Widowed	1483 (12.5%)	657 (12.9%)	826 (12.3%)	
Unknown	44 (0.4%)	17 (0.3%)	27 (0.4%)	
Race				
White or Caucasian	9583 (80.9%)	4262 (83.5%)	5321 (78.9%)	<0.001
Black or African American	1551 (13.1%)	601 (11.8%)	950 (14.1%)	
Other	713 (6.0%)	242 (4.7%)	471 (7.0%)	
Language				
English	9569 (80.8%)	4225 (82.8%)	5344 (79.3%)	<0.001
Other	2278 (19.2%)	880 (17.2%)	1398 (20.7%)	
Insurance				
Medicare	5432 (45.9%)	2584 (50.6%)	2848 (42.2%)	<0.001
Hospital	755 (6.4%)	256 (5.0%)	499 (7.4%)	
Medicaid	597 (5.0%)	134 (2.6%)	463 (6.9%)	
Private	2275 (19.2%)	821 (16.1%)	1454 (21.6%)	
Unknown/None	2788 (23.5%)	1310 (25.7%)	1478 (21.9%)	
Employment Status				
Employed	3441 (29.0%)	1347 (26.4%)	2094 (31.1%)	<0.001
Retired	5822 (49.1%)	2920 (57.2%)	2902 (43.0%)	
Unemployed	2315 (19.5%)	717 (14.0%)	1598 (23.7%)	
Unknown	82 (0.7%)	25 (0.5%)	57 (0.8%)	
Smoking Status				
Every Day	1006 (8.5%)	329 (6.4%)	677 (10.0%)	<0.001
Former	4727 (39.9%)	2047 (40.1%)	2680 (39.8%)	
Light Smoker	230 (1.9%)	79 (1.5%)	151 (2.2%)	
Never	5145 (43.4%)	2275 (44.6%)	2870 (42.6%)	
Unknown	104 (0.9%)	44 (0.9%)	60 (0.9%)	
Cancer Diagnosis				
Breast	3380 (28.5%)	1609 (31.5%)	1771 (26.3%)	<0.001
Benign	591 (5.0%)	201 (3.9%)	390 (5.8%)	
CNS	245 (2.1%)	70 (1.4%)	175 (2.6%)	
GI	752 (6.3%)	279 (5.5%)	473 (7.0%)	
GU	2284 (19.3%)	1160 (22.7%)	1124 (16.7%)	
GYN	723 (6.1%)	349 (6.8%)	374 (5.5%)	
HN	688 (5.8%)	386 (7.6%)	302 (4.5%)	
Lung	1432 (12.1%)	533 (10.4%)	899 (13.3%)	
Lymphoma	235 (2.0%)	68 (1.3%)	167 (2.5%)	
Misc	1061 (9.0%)	315 (6.2%)	746 (11.1%)	
Sarcoma	88 (0.7%)	43 (0.8%)	45 (0.7%)	
Skin	167 (1.4%)	92 (1.8%)	75 (1.1%)	
Charleson Comorbidity Index ≥ 4				
No	9201 (77.7%)	3875 (75.9%)	5326 (79.0%)	<0.001
Yes	2646 (22.3%)	1230 (24.1%)	1416 (21.0%)	
Charleson Comorbidity Index (Continuous)	3.00 (2.00-3.00)	3.00 (2.00-3.00)	2.00 (1.00-3.00)	<0.001
Encounter Type				
Follow-Up	8886 (75.0%)	3437 (67.3%)	5449 (80.8%)	<0.001
Consult	2675 (22.6%)	1666 (32.6%)	1009 (15.0%)	
OTV/Treatment	286 (2.4%)	2 (0.0%)	284 (4.2%)	
Telemedicine				
No	9351 (78.9%)	4081 (79.9%)	5270 (78.2%)	0.019
Yes	2496 (21.1%)	1024 (20.1%)	1472 (21.8%)	
Mental Health Disorder Type				
No Mental Health Disorder	10008 (84.5%)	4485 (87.9%)	5523 (81.9%)	<0.001
Anxiety	537 (4.5%)	205 (4.0%)	332 (4.9%)	
Behavioral Syndrome	20 (0.2%)	7 (0.1%)	13 (0.2%)	
Childhood Onset Behavioral/Emotion Disorder	18 (0.2%)	4 (0.1%)	14 (0.2%)	
Depression	288 (2.4%)	112 (2.2%)	176 (2.6%)	
Developmental Disorder	5 (0.0%)	3 (0.1%)	2 (0.0%)	
Intellectual Disability	3 (0.0%)	1 (0.0%)	2 (0.0%)	
Non-Mood Psychotic Disorder	24 (0.2%)	3 (0.1%)	21 (0.3%)	
Other Mood Disorder	53 (0.4%)	22 (0.4%)	31 (0.5%)	
Personality Disorder	3 (0.0%)	2 (0.0%)	1 (0.0%)	
Physiological Cause of Mental Disorder	41 (0.3%)	12 (0.2%)	29 (0.4%)	
Substance Use Disorder	847 (7.1%)	249 (4.9%)	598 (8.9%)	

Data presented as median (IQR) or n (%).

### Raw comparison of responders and non-responders

Of the total cohort, 5,105 participants (43.1%) were survey respondents, and 6,742 (56.9%) were non-respondents. In the raw comparison between survey respondents and non-respondents, shown in **Table 1**, significant differences emerged across several demographic and clinical characteristics. The median age of the overall cohort was 68 years, with respondents having a median age of 70 years and non-respondents having a median age of 66 years (p<0.001). Notably, 68.8% of respondents were over 65 years old, compared to 54.5% of non-respondents, indicating a significant age-related disparity (p<0.001). However, the gender distribution showed no significant difference, with females comprising 57.2% of respondents and 57.3% of non-respondents (p=0.917). The racial composition varied significantly; 83.5% of respondents were White or Caucasian, compared to 78.9% of non-respondents (p<0.001). Employment status also differed significantly, with 57.2% of respondents being retired compared to 43.0% of non-respondents (p<0.001). A significant difference was observed in language preference, with 82.8% of respondents primarily speaking English compared to 79.3% of non-respondents (p<0.001). Marital status showed disparities as well, with 60.6% of respondents being married compared to 56.1% of non-respondents (p<0.001).

In terms of cancer types, breast cancer was more prevalent among respondents (31.5%) than non-respondents (26.3%, p<0.001). Most respondents were never smokers (44.6%), compared to 42.6% of non-respondents (p<0.001). MHD were more common among non-respondents (18.1%) than respondents (12.1%, p<0.001), and a higher percentage of respondents had a CCI greater than 4 (24.1% vs. 21.0%, p<0.001).

A greater proportion of patients who attended consult visits completed the patient satisfaction survey (32.6%) compared to those with a consult visit but did not respond to the survey (15.0%, p<0.001). In contrast, fewer survey responders had follow-up visits (67.3%) compared to non-responders (80.8%, p<0.001). Additionally, the use of telemedicine was slightly more prevalent among non-respondents (21.8%) compared to respondents (20.0%), with a trend toward statistical significance (p=0.019). Insurance coverage also showed marked differences, with a higher percentage of respondents covered by Medicare (50.6%) and fewer having private insurance (16.1%) compared to 42.2% and 21.6% among non-respondents, respectively (p<0.001).

ADI data revealed substantial socioeconomic disparities, shown in **Table 2**, with a higher proportion of respondents living in areas with a national ADI rank below the mean (56.6% vs. 50.0%, p<0.001), indicating lower socioeconomic deprivation among respondents. Additionally, a higher proportion of respondents were in the first national ADI tertile (36.0%) compared to non-respondents (31.1%) (p<0.001).

**Table 2:** Comparison of patient engagement by Area Deprivation Index.

Characteristic	All Patients	Respondents	Non-Respondents	p value
National ADI Tertile				
1	3935 (33.2%)	1837 (36.0%)	2098 (31.1%)	<0.001
2	3756 (31.7%)	1668 (32.7%)	2088 (31.0%)	
3	3831 (32.3%)	1448 (28.4%)	2383 (35.3%)	
National ADI Above Mean				
No	6263 (52.9%)	2889 (56.6%)	3374 (50.0%)	<0.001
Yes	5584 (47.1%)	2216 (43.4%)	3368 (50.0%)	
National ADI Percentile	52.00 (31.00-75.00)	49.00 (30.00-71.00)	54.00 (33.00-77.00)	<0.001

Data presented as mean (range) or n (%).

ADI, Area Deprivation Index.

### Logistic regression analysis

**Figure 1:**
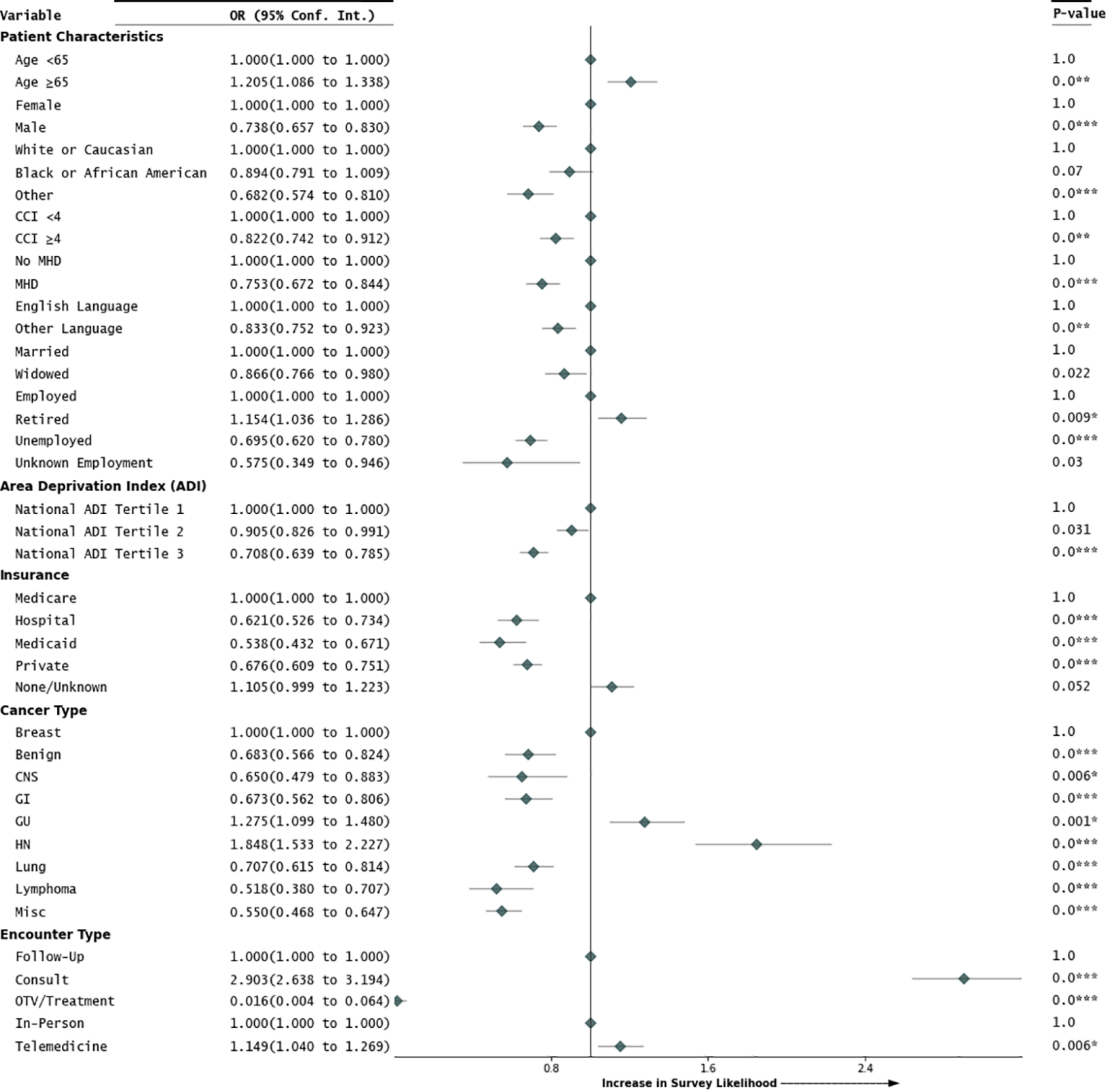
Multivariable analysis of factors impacting patient engagement. **Figure 1** presents a forest plot summarizing the multivariable analysis (MVA) of factors that influence patients’ likelihood of engagement in the patient satisfaction survey. It details odds ratios (OR) and their corresponding 95% confidence intervals (95% Conf. Int.) for each factor, with associated p-values displayed on the right side of the figure.

A UVA was first conducted to screen for significant and near-significant predictors to include in the MVA backward stepwise logistic regression model, examining a range of factors potentially influencing patient satisfaction survey response. All variables included in the MVA were significant or near significant in the UVA. In the MVA, outlined as a forest plot in **Figure 1**, SES, as indicated by the ADI, emerged as a significant determinant of survey participation. Specifically, patients in the second national ADI tertile exhibited a modest trend toward a significant decrease in likelihood of survey response (OR=0.905, 95% CI=0.826–0.991, p=0.031) compared to the first tertile. This trend was more pronounced in the third national ADI tertile, where patients experienced a more substantial decline in survey engagement (OR=0.708, 95% CI=0.639–0.785, p<0.001), suggesting that people facing higher levels of socioeconomic disadvantage may be less likely to engage in surveys. A separate MVA model was conducted with national ADI as a continuous variable and indicated that a 1 unit increase in ADI was associated with a 1% reduction in the odds of survey completion (p<0.001).

The propensity to participate in the patient satisfaction survey was significantly higher among individuals older than 65 (OR=1.205, 95% CI=1.086–1.338, p<0.001). Regarding gender dynamics, male patients demonstrated a lower likelihood of survey completion, indicating a potential gender gap in patient engagement (OR=0.738, 95% CI=0.657–0.830, p<0.001). Racial disparities in patient satisfaction survey engagement were partially observed; Black or African American patients showed no significant difference in likelihood of response compared to White patients (OR=0.894, 95% CI=0.791–1.009, p=0.07). However, other minorities were significantly less likely to engage with the patient satisfaction surveys when compared to White patients (OR=0.682, 95% CI=0.574–0.810, p<0.001). The *post-hoc* analysis showed that there was a higher proportion of minority groups classified as ‘Other’ in the first ADI tertile (67.5%) compared to White patients in the first ADI tertile (56.1%), with the difference being statistically significant (p<0.001).

Language also played a role, with patients who primarily spoke languages other than English being less likely to participate (OR=0.833, 95% CI=0.752–0.923, p<0.01). Marital status affected engagement, with widowed patients showing a lower likelihood of participation compared to married patients (OR=0.866, 95% CI=0.766–0.980, p=0.022). Employment status was another significant factor; retired patients were more likely to complete surveys compared to employed individuals (OR=1.154, 95% CI=1.036–1.286, p=0.009), while unemployed patients were less likely to engage (OR=0.695, 95% CI=0.620–0.780, p<0.001).

Cancer type significantly influenced the likelihood of survey participation. Head and neck cancer patients were almost twice as likely to respond to the patient satisfaction survey (OR=1.848, 95% CI=1.533–2.227, p<0.001) compared to breast cancer patients. Genitourinary cancer patients had a higher response propensity (OR=1.275, 95% CI=1.099–1.480, p=0.001), while gastrointestinal (OR=0.673, 95% CI=0.562–0.806, p<0.001) and lymphoma patients (OR=0.518, 95% CI=0.380–0.707, p<0.001) were less likely to respond. Patients with benign diagnoses (OR=0.683, 95% CI=0.566–0.824, p<0.001), central nervous system cancers (OR=0.650, 95% CI=0.479–0.883, p=0.006), and lung cancers (OR=0.707, 95% CI=0.615–0.814, p<0.001) were also less likely to engage. Additionally, patients with a CCI score of 4 or more were significantly less likely to respond (OR=0.822, 95% CI=0.742–0.912, p<0.001), indicating health status affects patient engagement.

Consultation encounters were strongly correlated with increased survey completion (OR=2.903, 95% CI=2.638–3.194, p<0.001), while treatment visits had a reduced response likelihood (OR=0.016, 95% CI=0.004–0.064, p<0.001) compared to follow-up visits. Interestingly, telemedicine encounters showed significantly higher survey engagement (OR=1.149, 95% CI=1.040–1.269, p=0.006) compared to in-person encounters. A *post-hoc* Pearson’s chi-square test indicated that patients in the third ADI tertile used telemedicine more (64.2% vs. 45.3% in-person, p<0.001). Men were also more likely to engage in telemedicine (25.8% vs. 17.1% for women, p<0.001).

Insurance type emerged as a key factor in determining survey response rates. Patients with hospital insurance (OR=0.621), Medicaid (OR=0.538), and private insurance (OR=0.676) were less likely to complete surveys than those on Medicare (all p-values <0.001). The *post-hoc* Kruskal-Wallis test showed the median age for Medicare patients was 73 years (IQR: 69–78), significantly higher than other insurance types at 61 years (IQR: 54–67), (p<0.001).

The likelihood of patient engagement in satisfaction surveys was also significantly lower among patients with MHD (OR=0.753, 95% CI=0.672–0.844, p<0.001). A separate MVA, outlined in **Supplementary Figure 1**, explored the specific types of MHD, using “No MHD” as the reference group. This analysis highlighted that patients with substance use disorders (OR=0.671, 95% CI=0.569–0.792, p<0.001) were significantly less likely to engage with the survey. Patients with non-mood psychotic disorders (OR=0.214, 95% CI=0.061–0.743, p=0.015) showed a trend toward a significant reduction in their likelihood for patient engagement of the patient satisfaction survey. Patients with MHD were also more likely to fall into the third ADI tertile (57.0%) compared to those without an MHD (47.9%, p<0.001).

## Discussion

This extensive retrospective study has identified multiple factors that influence patient engagement in satisfaction surveys within the radiation oncology patient population.

### Socioeconomic status

Our investigation into the relationship between SES and patient engagement in satisfaction surveys has uncovered a clear and direct connection: as socioeconomic deprivation increases, patient participation in surveys declines. Notably, each unit rise in the ADI national percentile rank is associated with a 1% decrease in the odds of survey completion. This trend is alarming as it implies that crucial patient feedback may be overlooked, especially from those in lower SES brackets, potentially leading to an incomplete understanding of patient dissatisfaction.

Komaromy et al. (31) highlighted that factors eroding trust and engagement among lower SES patients include limited availability for thorough interactions, perceived judgmental attitudes, and an inability to address essential needs. Addressing these areas is essential for enhancing engagement and ensuring that patient satisfaction surveys reflect the experiences of all societal segments (31).

The broader implications of SES on health outcomes are well-documented. Lower SES is often a precursor to poorer health behaviors and a higher incidence of all-cause mortality, as well as shaping patients’ perceptions of clinical bias and reducing satisfaction with care (32–35). Given that patient dissatisfaction is intricately linked with significant operational metrics—ranging from hospital profitability to readmission rates—the observation of reduced patient engagement in conjunction with higher SES disadvantage resonates with prior findings and underscores the pressing need for targeted intervention (7–13).

In this context, innovative strategies are imperative to mitigate the effects of SES on patient engagement. A study by Bull et al. (36) provides a promising example, where telephone health coaching delivered over several months resulted in reduced mortality among older, low-income men (36). Although this benefit was not universal across all demographics, it suggests effective engagement avenues for underrepresented populations. Continuing to develop and implement such interventions is crucial for fostering inclusive patient feedback, ultimately guiding healthcare improvements that benefit all patient groups.

### Age

Our findings show that individuals over 65, as well as retired patients, are more inclined to participate in surveys than younger patients. This pattern aligns with literature suggesting that older adults have more positive attitudes and altruistic motivations toward survey completion, as documented by von Strauss et al. (37) and Fiordelli et al. (38). Despite common barriers to clinical trial participation for older individuals, such as a lack of familiarity with research processes or limited access to information about participation, as well as feeling overwhelmed by health issues (39, 40), satisfaction surveys appear less demanding and immediately relevant, making them easier to complete (39–41).

In our examination of insurance types and their influence on survey participation, a pattern emerged: Medicare, used as the comparator group, had a significantly higher likelihood of patient engagement than any other insurance type, paralleling the trends seen with advancing age. Since Medicare predominantly serves individuals aged 65 and above, this insurance variable dovetails with the increased likelihood of survey engagement noted in older populations. Despite low multicollinearity in the MVA model, Medicare coverage may act as a proxy for age in influencing patient engagement.

In contrast, the relationship between insurance type and patient engagement in our study differs from Theiss et al. (42), where privately insured patients had higher engagement with patient-reported outcomes surveys (42). While their cohort’s median age was 66, comparable to ours, only 5.7% of their participants were covered by Medicare, leading to a marked discrepancy in representation (42). This discrepancy suggests that the higher engagement among privately insured patients in their study may not be directly comparable to our findings, highlighting the importance of demographic representation when interpreting the drivers of patient engagement.

### Race and gender

Despite similar representation of male and female responders in the Pearson’s chi-square test, the MVA indicated significant gender-based disparities, with male patients being less likely to complete the survey. This aligns with existing studies by Etingen et al. (43) and Höhn et al. (44), which show higher utilization of health services by women, along with greater patient engagement and activation (43, 44). Novak et al. (45) identified traditional masculine norms as a barrier to healthcare use for heteronormative men (45). Bidmon et al. (46) also noted that women were more proactive in seeking health information online and placed greater importance on social and interactive web features, despite self-reported lower digital competence than men (46). Interestingly, our study found a higher frequency of male patients using telemedicine encounters than female patients, suggesting that men may be less inclined toward traditional satisfaction surveys but are more engaged in digital telemedicine. This emphasizes the need for tailored strategies to improve patient engagement across different communication channels.

Race also revealed intriguing patterns of survey engagement within our cohort. Unlike the findings by Eliacin et al. (47), where Black patients showed lower patient activation and engagement than White patients, our study found no significant difference in survey response rates between Black and White patients (47). However, a stark difference was noted among patients from other minority groups, who were less likely to engage in surveys. A *post-hoc* analysis revealed that a larger proportion of ‘Other’ minorities were in the least deprived SES tertile compared to White patients, suggesting that factors beyond SES affect survey participation.

Language barriers may significantly impact survey participation among ‘Other’ minority groups (25). Rittase et al. (48) found that English web-based surveys had higher response rates than Spanish ones, especially among nonwhite, lower-income, or less-educated individuals (48). Moreover, Carrasquillo et al. (49) found that non-English speakers in emergency departments reported lower satisfaction, reduced willingness to return, and more problems with care compared to English speakers (49). Our study found that patients who had a preferred language other than English were less likely to participate in the surveys, despite the use of multi-language NRC patient satisfaction surveys. Future studies should aim to improve survey participation among non-English speakers by addressing these language barriers.

### Cancer type and comorbidities

Our analysis revealed that patients with a CCI score above 4 were less likely to engage in satisfaction surveys, consistent with findings by Hibbard et al. (4), which highlighted how comorbidities impact patient involvement (4). To mitigate this, Wong et al. (50) suggested that increased provider-patient interaction significantly enhances patient engagement patients, recommending more intensive communication to improve participation from these patients (50).

Among cancer types, patients with head and neck cancers were the most likely to participate in our surveys. Their increased engagement may be due to the acute complications and long-term consequences these patients face, requiring enhanced care and support. Pompili et al. (51) observed that patients experiencing complications often report higher satisfaction, likely due to the increased attention and personalized care they receive (51).

In contrast, patients with benign diseases or those with central nervous system, lung, gastrointestinal, and lymphoma cancers were less likely to engage in satisfaction surveys These disparities are likely complex and disease-specific, potentially influenced by disease severity and the quality of provider interactions. Further investigation is warranted to identify barriers and develop targeted strategies to improve participation across these cancer types.

### Healthcare utilization

Interestingly, telemedicine appointments exhibited a trend toward increased participation in satisfaction surveys, with a *post-hoc* analysis revealing higher telemedicine use among the most socioeconomically deprived patients. This finding suggests telemedicine’s accessibility, particularly through mobile apps, may reduce the negative impact of low SES on patient engagement. The seamless transition from a telemedicine encounter to completing surveys on the same digital device is hypothesized to boost survey completion rates. This concept of leveraging technology to enhance patient enhancement is supported by various studies, including randomized controlled trials (52–56). However, a recent randomized controlled trial identified a decrease in patient activation associated with mobile app use in prenatal care, suggesting the need for a nuanced approach (57). The variability in mobile app effectiveness across different patient populations highlights the necessity of tailored digital health interventions.

Comparative studies like Darrat et al. (58) show varying results on the relationship between telemedicine and SES, with lower SES head and neck surgical patients during the COVID-19 pandemic less likely to complete telemedicine encounters (58). These differences could stem from differences in study conditions, timeframes, and socioeconomic measures. Our study, which used the ADI for SES measurement and included a broader range of cancer types in a radiation oncology setting, also utilized diverse communication methods like email, IVR, and SMS to maximize patient outreach. Mobile apps have the potential to further enhance patient engagement, particularly among follow-up patients. Ongoing research by Kwan et al. (59) on using mobile apps for patient engagement and activation in type 2 diabetes management could provide additional insights (59).

### Mental health

MHD have a notable impact on patient engagement in satisfaction surveys, particularly among individuals with substance use disorders, who are significantly less likely to participate. This pattern may be due to various factors, including stigma or challenges accessing healthcare. Moreover, patients with anxiety and non-mood psychotic disorders exhibit a trend toward reduced engagement. A *post-hoc* analysis found that patients with MHD were more likely to fall into higher ADI tertiles, suggesting a correlation between socioeconomic disadvantage and MHD that further complicates survey participation.

MHD has been linked to social isolation, and widowed patients often face additional challenges (60, 61). Despite Isherwood et al. (62) showing higher social engagement in widowed adults, they suggest that those in lower socio-economic groups, in poorer health, or without a child living nearby were found to have lower levels of social engagement in widowhood, and may be at increased risk of social isolation (62). Our study showed a significantly lower likelihood of engaging in the satisfaction survey among these groups. Perhaps increased social engagement would help improve participation.

Research highlights the potential of digital interventions in improving patient engagement among those with mental health conditions. A randomized controlled trial by McCue et al. (63) found that mobile app usage for symptom tracking in patients with major depressive disorder led to significant long-term improvements in patient activation and a numerical, though not statistically significant, improvement in engagement (63). Similarly, a randomized controlled trial by Vitger et al. (55) showed that using a smartphone app in patients with non-mood psychotic disorders, such as schizophrenia, can increase patient activation and support shared decision-making in outpatient settings (55).

These findings highlight the need for innovative strategies to engage patients with MHD in healthcare feedback. By incorporating targeted digital tools and personalized approaches, healthcare providers can enhance the inclusivity and efficacy of patient satisfaction surveys, ensuring that the voices of all patient groups are heard and considered.

### Limitations

This study, while large and novel, faces several limitations that warrant consideration. The retrospective design inherently limits the ability to infer causality from observed associations, underscoring the need for prospective studies to clarify these findings. Reliance on administrative data like ICD-10 coding for capturing MHD and CCI might not fully reflect the clinical nuances of each patient’s health profile, affecting the precision of the associations measured. Additionally, different survey modalities, such as email, SMS, and IVR, were not independently evaluated for their impact on response rates, potentially limiting insights. The study was conducted at an academic center, which may influence the results and not necessarily capture the full breadth of demographic trends observed in private practice, community centers, and rural practices. Furthermore, the effect of treatment visits on survey participation is likely an artifact, as patients who completed a survey during a consultation were not surveyed again for the next 120 days, systematically reducing the likelihood of treatment visit responses. Finally, COVID-19’s temporal changes could have influenced patient attitudes toward telemedicine and healthcare engagement. As patient engagement strategies and their efficacy evolve over time, addressing these limitations is essential to advancing our understanding of patient engagement and improving patient-centered care.

### Future directions

Healthcare disparities have gained increasing recognition in radiation oncology and medicine as a whole, emerging as a critical area of research. These disparities encompass inequities in access to care, treatment outcomes, and patient experience, particularly among socioeconomically disadvantaged populations. This study highlights the need for actionable strategies to enhance patient engagement and satisfaction. Addressing socioeconomic barriers involves implementing targeted outreach programs, culturally inclusive communication materials, and offering survey options in multiple languages. Improving telemedicine and mobile health platforms to ensure equitable access and user-friendliness can help close participation gaps, particularly among underrepresented groups. Survey data should inform tangible changes in clinical practice, such as creating tailored support services for patients with MHD or addressing disparities in care access. Future prospective studies are crucial to validate these findings and assess the impact of these initiatives on patient engagement.

## Conclusion

As the largest and most comprehensive retrospective study of its kind, this research provides an in-depth examination of patient engagement within the radiation oncology patient population. Drawing on our extensive data, it is evident that a confluence of factors influences patient engagement in satisfaction surveys. Individuals of higher socioeconomic status, telemedicine visits, older patients with Medicare insurance, and females are more apt to participate in feedback mechanisms. Contrastingly, the likelihood of engagement diminishes among patients grappling with mental health disorders, those burdened with significant comorbidities, widowed patients, and members of minority groups. These findings underscore the importance of tailored approaches to capture a broader and more representative spectrum of patient experiences, ensuring that the insights gleaned from satisfaction surveys truly reflect the diverse patient population we serve.

## Data Availability

The raw data supporting the conclusions of this article will be made available by the authors, without undue reservation.
