# Supplementary material for: Patient engagement in radiation oncology: a large retrospective study of survey response dynamics
Source: Front Oncol. 2025 Jan 17;14:1434949. doi: 10.3389/fonc.2024.1434949 (PMC11782270; doi:10.3389/fonc.2024.1434949)
Supplement: Supplementary file 1 [file DataSheet1.docx]

Supplementary Material

## Supplementary Figures

**Supplemental Table 1. The NRC PS survey**

| **No** | **Survey Question Topic** | **Question Description** |
| --- | --- | --- |
| 1 | Clerk/Receptionist: Courtesy & Respect | Did clerks and receptionists at this provider’s office treat you with courtesy and respect? |
| 2 | Nurse: Adequate Explanation | Did nurse explain things in a way you could understand? |
| 3 | Nurse: Listened | Did nurse listen carefully to you? |
| 4 | Nurse: Courtesy & Respect | Did nurse treat you with courtesy and respect? |
| 5 | Team Member: Listened | Did team members listen carefully to you? |
| 6 | Doctor: Adequate Explanation | Did we explained things in a way you could understand? |
| 7 | Nurse: Confidence & Trust | Did you have confidence and trust in the nurses treating you? |
| 8 | Doctor: Enough Input in Care | Did you have enough input or say in your care? |
| 9 | Nurse Present | Did you see a nurse during this visit? |
| 10 | Overall: Recommend Facility | How likely would you be to recommend this facility to your family and friends? |
| 11 | Overall: Recommend Provider | How likely would you be to recommend this provider to your family and friends? |
| 12 | Family or Friend Involved | Was a family member or friend allowed to be involved in your treatment as much as you wanted? |
| 13 | Nurse/Doctor: Interprofessional Communication | Was there good communication between the different doctors and nurses? |
| 14 | Nurse: Comfortable Talking | Were you comfortable talking with nurses about your worries or concerns? |

**Supplemental Table 2. Codes and scales used in the National Research Council patient satisfaction survey**

| Likert Scale for Questions 1-8, 12-14 | |
| --- | --- |
| 0 | No answer |
| 1 | No |
| 2 | Yes, somewhat |
| 3 | Yes, mostly |
| 4 | Yes, definitely |
| 5 | Not applicable |
| Scale for Questions 10-11 | |
| 0-10 | Scale |
| Codes for Question 9 | |
| 0 | No answer |
| 1 | No |
| 2 | Yes |

**Supplementary Figure 1. Multivariable Analysis of Factors Impacting Patient Engagement Subgrouping Mental Health Disorders**


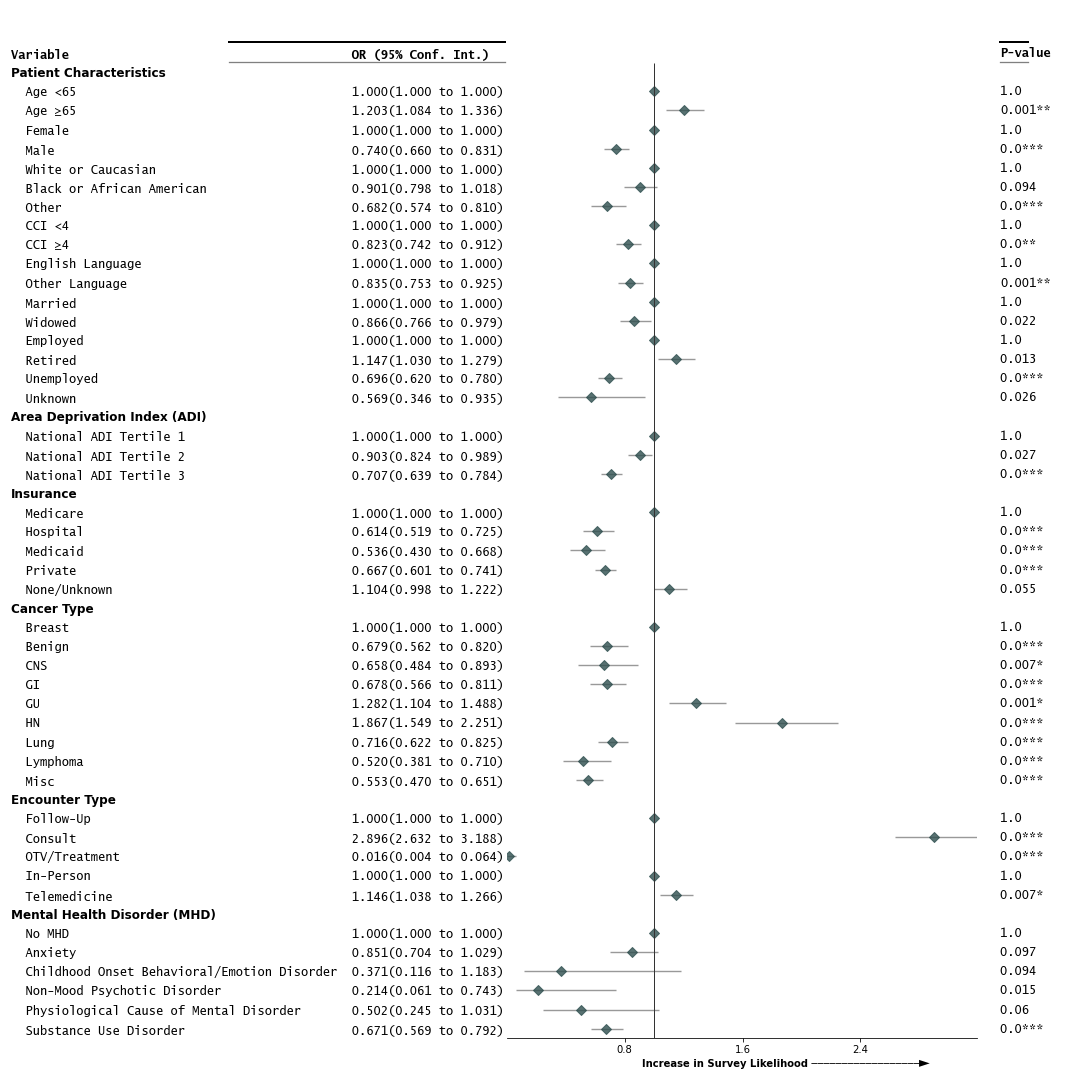


Supplementary Figure 1 presents a forest plot summarizing the multivariable analysis (MVA) of factors influencing patients' likelihood of engagement in the patient satisfaction survey. This model replaces the binary mental health disorders (MHD) variable with specific mental health disorder types, including substance use disorders and non-mood psychotic disorders. It provides odds ratios (OR) with 95% confidence intervals (95% Conf. Int.) for each factor, alongside their p-values, to indicate the strength and significance of associations. The analysis used the same backward stepwise logistic regression method as the primary MVA, with "No MHD" as the reference group.
